# An Accessible AI-Assisted Rehabilitation System for Guided Upper Limb Therapy

**DOI:** 10.3390/s25196239

**Published:** 2025-10-08

**Authors:** Kevin Hou, Md Mahafuzur Rahaman Khan, Mohammad H. Rahman

**Affiliations:** 1 Brookfield Central High School, Brookfield, WI 53005, USA; kevinyadihou@gmail.com; 2 Department of Mechanical Engineering, University of Wisconsin-Milwaukee, Milwaukee, WI 53211, USA; rahmanmh@uwm.edu

**Keywords:** upper limb rehabilitation, computer vision, pose detection, home-based rehabilitation, telerehabilitation

## Abstract

Conventional upper limb rehabilitation methods often encounter significant obstacles, including high costs, limited accessibility, and reduced patient adherence. Emerging technological solutions, such as telerehabilitation, virtual reality (VR), and wearable sensor-based systems, address some of these challenges but still face issues concerning supervision quality, affordability, and usability. To overcome these limitations, this study presents an innovative and cost-effective rehabilitation system based on advanced computer vision techniques and artificial intelligence (AI). Developed using Python (3.11.5), the proposed system utilizes a standard webcam in conjunction with robust pose estimation algorithms to provide real-time analysis of patient movements during guided upper limb exercises. Instructional exercise videos featuring an NAO robot facilitate patient engagement and consistency in practice. The system generates instant quantitative feedback on movement precision, repetition accuracy, and exercise phase completion. The core advantages of the proposed approach include minimal equipment requirements, affordability, ease of setup, and enhanced interactive guidance compared to traditional telerehabilitation methods. By reducing the complexity and expense associated with many VR and wearable-sensor solutions, while acknowledging that some lower-cost and haptic-enabled VR options exist, this single-webcam approach aims to broaden access to guided home rehabilitation without specialized hardware.

## 1. Introduction

Musculoskeletal disorders significantly contribute to the global burden of disability, affecting approximately 1.7 billion individuals worldwide and representing the leading cause of long-term impairment [1]. Disorders involving the upper limb are particularly debilitating, as they severely restrict essential daily activities. The causes behind upper limb impairments are numerous and include repetitive strain injuries due to occupational overuse, neurological impairments from conditions like stroke, acute traumas such as fractures, and degenerative diseases like osteoarthritis [1,2,3,4]. Among these, stroke is notably prevalent, often leaving survivors with persistent motor deficits in the upper extremities [3]. Beyond stroke, upper limb motor impairments also arise in cerebral palsy [5], Parkinson’s disease, and multiple sclerosis, where structured, repetitive practice can improve range of motion, strength, and task ability [6,7,8]. Including these populations clarifies the general need for accessible, feedback-rich home exercise. Across these populations, structured rehabilitation improves upper limb outcomes: systematic reviews on Parkinson’s disease show benefits for hand dexterity and function, reviews on multiple sclerosis support effects of upper limb training, and CP guidelines synthesize effective motor treatments [6,9,10].

Despite the high incidence and substantial impact of upper limb impairments, considerable barriers remain regarding accessibility, affordability, and patient adherence to rehabilitation programs. In low-resource settings, geographical barriers, inadequate availability of qualified therapists, and prohibitive treatment costs severely restrict access to rehabilitation services. Alarmingly, it has been estimated that 90% of individuals in low-income countries requiring rehabilitation do not receive appropriate care [11]. Even where rehabilitation is available, long-term patient engagement remains problematic. Rates of non-adherence to prescribed home exercise programs are notably high, ranging from 50% to 70% among individuals with musculoskeletal conditions [12]. Consequently, these significant challenges markedly diminish the overall efficacy of conventional rehabilitation strategies.

Technological interventions, including virtual reality (VR), wearable sensors, and telerehabilitation via video conferencing, have been explored to address these barriers. However, each approach has critical shortcomings. VR platforms can deliver immersive practice with rich feedback, and some systems incorporate haptic/force cues; entry-level VR has also reduced financial barriers [13]. In parallel, low-cost VR and exergaming options (e.g., consumer HMD/serious-game systems; Nintendo® Wii-based programs in CP) have reduced entry barriers compared with those for clinic-grade rigs [5,14]. Nevertheless, these typically require specialized hardware and setup, which can limit deployment in low-resource homes. Wearable sensors (e.g., IMUs) offer illumination independence and robustness to occlusion while maintaining excellent portability, remaining independent of ambient illumination and line-of-sight occlusion; however, they require additional devices for donning and doffing [15,16]. Our goal is a minimal-equipment path that complements these approaches when sensors or head-mounted displays are not practical [17]. Wearable sensors such as inertial measurement units and sensor-integrated gloves provide accurate motion tracking but require users to manage additional hardware, potentially reducing usability and adherence [18]. Likewise, conventional telerehabilitation through video calls expands accessibility but fails to deliver the precise, real-time feedback necessary for adequate exercise supervision [19]. Hence, a clear need persists for rehabilitation solutions that combine technological accessibility, minimal equipment requirements, and interactive, real-time feedback.

This work’s novelty is the integration of three elements into one accessible, single-webcam platform: (i) per-session, individualized range of motion calibration used to qualify repetitions; (ii) socially assistive guidance via short, standardized NAO demonstrations; and (iii) real-time quantitative feedback, including a within-session accuracy analysis that summarizes the average repetition and reports the RMSE relative to a didactic target [20,21]. We then evaluate the system by validating the angle accuracy against a reference setup and by assessing the within-session performance change and user feedback in home-style trials.

## 2. Background Study

Markerless computer vision has emerged as a highly effective approach to rehabilitation monitoring, eliminating the need for wearable sensors or markers [22]. Pose estimation technologies, notably OpenPose [23] and MediaPipe [24], facilitate the real-time detection and tracking of skeletal landmarks using standard webcams. These technologies provide clinicians with accurate joint angle measurements and range of motion analyses, offering a cost-effective and accessible alternative to traditional motion capture systems. Previously prevalent hardware-based solutions, such as Microsoft’s Kinect, have gradually been replaced by simpler, webcam-based alternatives tailored to home-based rehabilitation scenarios [22]. Validation studies support the efficacy of these markerless approaches, demonstrating accuracy comparable to that of traditional motion-capture systems for assessing various rehabilitation exercises, including squats, and gait analyses [25,26].

Despite these advantages, single-camera pose estimation presents certain limitations, particularly concerning depth ambiguity and occlusion. A single 2D perspective limits accurate tracking of movements toward or away from the camera, potentially reducing measurement precision [27]. Occlusion—occurring when limbs overlap—further complicates accurate joint detection [25]. To address these challenges, advancements such as 3D pose estimation methods involving multi-camera configurations or depth estimation algorithms, such as MediaPipe’s BlazePose, have been proposed [24]. Additionally, careful patient positioning and exercise orientation can mitigate occlusion issues, enhancing the reliability of single-camera setups. For example, fixed frontal or lateral camera placements have been successfully utilized in stroke rehabilitation to maximize limb visibility and tracking accuracy [28].

Beyond motion tracking alone, rehabilitation effectiveness is also significantly enhanced by visual guidance provided by humanoid robots and virtual avatars. Socially assistive robots, exemplified by the NAO robot from SoftBank Robotics, contribute positively to patient motivation and compliance through engaging physical demonstrations combined with verbal encouragement [29,30]. Research by Raso et al. demonstrated improved smoothness and temporal control of shoulder rehabilitation exercises when guided by a NAO robot compared to traditional approaches [30]. Similarly, virtual avatars used in digital coaching platforms have effectively instructed patients, demonstrating comparable clinical outcomes and improved compliance relative to conventional methods [31].

Integrating these technologies, recent home-based telerehabilitation systems have leveraged real-time feedback via markerless pose estimation. Adolf et al. introduced an OpenPose-based system that effectively tracked joint movements during home exercises, albeit with occasional accuracy reductions in certain positions such as floor-based exercises [25]. Cóias et al. developed a webcam-assisted virtual coaching system specifically targeting stroke rehabilitation, successfully providing real-time audiovisual feedback to correct excessive trunk compensatory movements [28]. These systems have been validated for efficacy and patient acceptability, particularly critical during increased remote rehabilitation adoption driven by the COVID-19 pandemic [31].

Nevertheless, current systems continue to rely heavily on specialized hardware or exhibit limited real-time responsiveness, particularly when deployed on low-powered devices, thereby affecting accessibility and usability [22,27,28]. As summarized in Table 1, a range of prior work on webcam-based systems, humanoid robots, and wearables has highlighted specific limitations such as occlusion and setup complexity. Additionally, purely virtual coaching solutions often lack the physical interaction and motivational presence provided by humanoid robots. Addressing these gaps, the proposed system integrates real-time pose estimation using MediaPipe, demonstrative guidance from the NAO robot, and immediate, automated corrective feedback. This innovative approach provides an affordable, user-friendly, and scalable rehabilitation solution suitable for widespread deployment in home settings, aiming to significantly enhance patient compliance and overall rehabilitation outcomes.

## 3. Methodology

### 3.1. Exercise Selection and NAO Robot Demonstration

Four exercises targeting the shoulder and elbow joints were selected based on clinical evidence, relevance, and documented effectiveness in enhancing functional recovery of upper limb impairments. The selected exercises included.

**Shoulder Abduction and Adduction:** These exercises strengthen the deltoid and supraspinatus muscles, improving shoulder stability, and are fundamental for restoring the ability to perform lateral reaching. They are especially important for patients recovering from conditions such as rotator cuff injuries, adhesive capsulitis, and hemiparesis following stroke [35,36]. Additionally, these movements enhance scapulohumeral rhythm, reduce muscular imbalance, and prevent joint dysfunction [37].

**Shoulder Flexion and Extension:** Crucial for activities such as forward-reaching, overhead lifting, and pushing/pulling movements required in daily life; incorporation of these exercises is critical following adhesive capsulitis, tendonitis, and fractures involving the proximal humerus, all of which lead to limitations in independence. Regular execution significantly improves range of motion (ROM), reduces pain, and accelerates functional rehabilitation [36,38].

**Elbow Flexion and Extension:** Essential for enhancing joint mobility and strength, these exercises help manage conditions such as post-fracture stiffness, tendinopathies, and stroke-related impairments, aiding in reductions in stiffness and spasticity and improving daily function [38,39].

**Shoulder External and Internal Rotation:** These movements play a vital role in rotator cuff rehabilitation, shoulder joint stability, and the management of conditions like adhesive capsulitis. Properly structured rotational exercises effectively reduce shoulder pain and enhance functional ROM [36,37].

**Figure 1:**
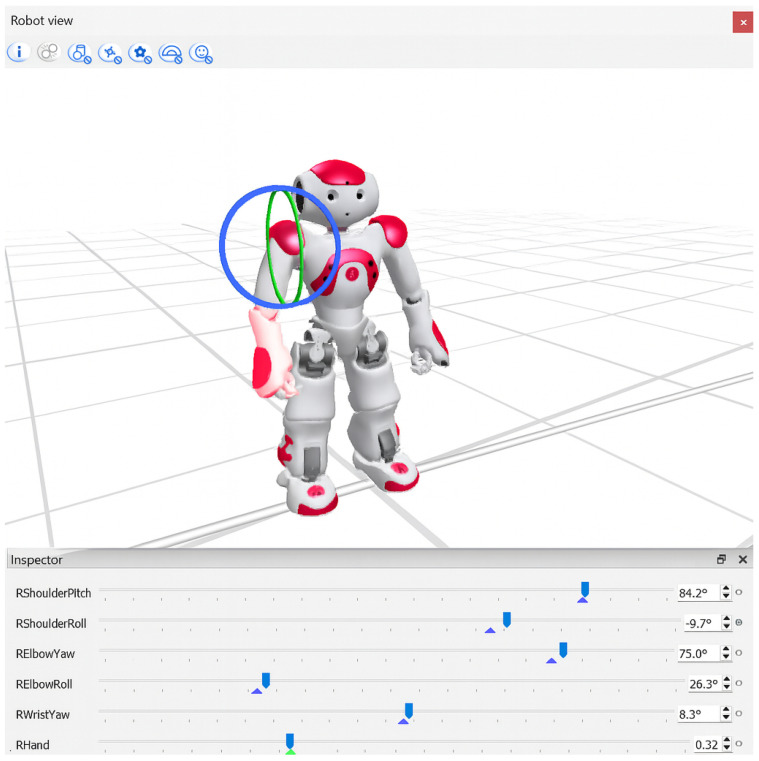
The virtual NAO robot inside Choregraphe where the selected joint can be manipulated to a specified degree.

To facilitate patient engagement, each exercise was visually demonstrated by the humanoid NAO robot (SoftBank Robotics), shown in Figure 1. Demonstration motions were developed using Choregraphe’s timeline editor, precisely defining the keyframes for initial, intermediate, and final exercise positions. Intermediate positions were automatically interpolated, resulting in smooth, natural motions. While the demonstrations were informed by exercises documented in the clinical literature, they were not directly supervised or formally validated by physiotherapists, which we recognize as a limitation of this study. Robot demonstrations were captured as short video clips (10–15 s) and integrated within the rehabilitation software, providing clear visual instructions that patients could easily follow [30,40].

### 3.2. Pose Estimation and Joint Angle Calculations

**Figure 2:**
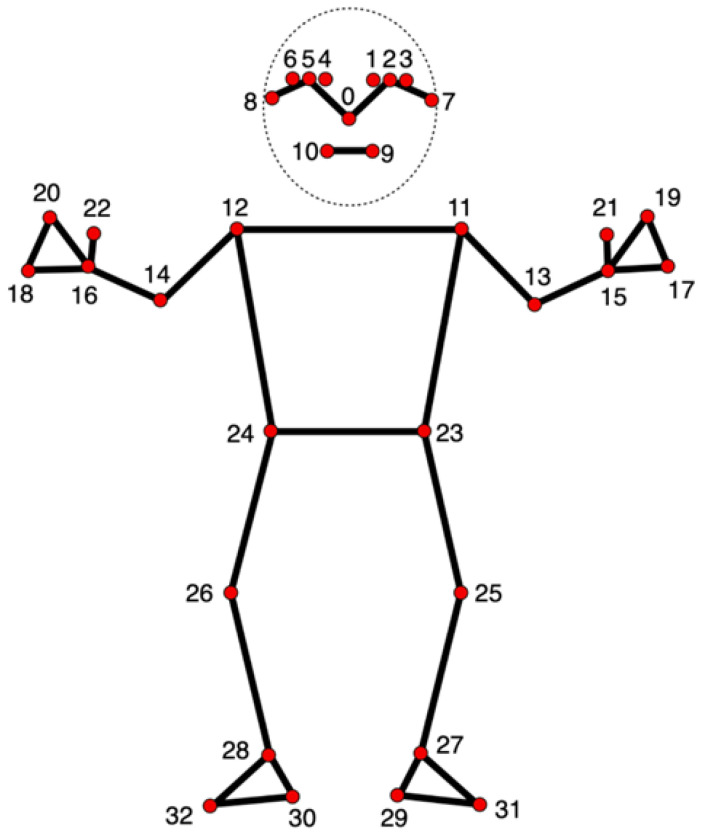
All 33 landmarks tracked by the MediaPipe Pose model.

The system utilizes MediaPipe Pose (landmark map shown in Figure 2) to detect critical anatomical landmarks in real time from the webcam input, a framework known for high accuracy in human pose tracking [24]. Joint angles were computed using vector mathematics based on the extracted landmark coordinates, with each equation described in order below. All variables are defined explicitly to ensure reproducibility.

#### 3.2.1. General Joint Angle Calculation

Given three anatomical landmarks represented by points $A(x_{a},y_{a})$, $B(x_{b},y_{b})$, and $C(x_{c},y_{c})$, the vectors $V_{1}$ and $V_{2}$ are defined as

(1) $$\begin{array}{cc} V_{1} & =\vec{BA}=\left(x_{a}-x_{b},\;y_{a}-y_{b}\right), \end{array}$$

(2) $$\begin{array}{cc} V_{2} & =\vec{BC}=\left(x_{c}-x_{b},\;y_{c}-y_{b}\right). \end{array}$$

The dot product of the two vectors is

(3) $$V_{1}\cdot V_{2}=\left(x_{a}-x_{b}\right)\left(x_{c}-x_{b}\right)+\left(y_{a}-y_{b}\right)\left(y_{c}-y_{b}\right),$$

and their magnitudes are

(4) $$\begin{array}{cc} \left|\right|V_{1}\left|\right| & =\sqrt{{\left(x_{a}-x_{b}\right)}^{2}+{\left(y_{a}-y_{b}\right)}^{2}}, \end{array}$$

(5) $$\begin{array}{cc} \left|\right|V_{2}\left|\right| & =\sqrt{{\left(x_{c}-x_{b}\right)}^{2}+{\left(y_{c}-y_{b}\right)}^{2}}. \end{array}$$

The angle θ at joint *B* is then given by the cosine rule:

(6) $$\theta =\cos^{-1}\;\left(\frac{V_{1}\cdot V_{2}}{\left|\right|V_{1}\left||\;|\right|V_{2}\left|\right|}\right).$$

This general approach was used for shoulder abduction/adduction, shoulder flexion/extension, and elbow flexion/extension.

#### 3.2.2. Shoulder Medial/Lateral Rotation

Direct measurement of shoulder medial/lateral rotation is difficult using a 2D camera setup. Instead, the forearm was used as an indicator, with correction for perspective distortion. The forearm vector is defined as

(7) $$v_{\mathrm{forearm}}=\left(w_{x}-e_{x},\;w_{y}-e_{y}\right),$$

where $\left(e_{x},e_{y}\right)$ and $\left(w_{x},w_{y}\right)$ are the elbow and wrist coordinates, respectively.

The forearm length is

(8) $$|v_{\mathrm{forearm}}|=\sqrt{{\left(w_{x}-e_{x}\right)}^{2}+{\left(w_{y}-e_{y}\right)}^{2}}.$$

Because elbow flexion alters the perceived forearm length in 2D, a correction factor was applied:

(9) $$L_{\mathrm{corrected}}=\left|v_{\mathrm{forearm}}\right|\cdot \sin\left(\theta_{\mathrm{elbow}}\right),$$

where $\theta_{\mathrm{elbow}}$ is the elbow joint angle, defined as

(10) $$\theta_{\mathrm{elbow}}=\cos^{-1}\;\left(\frac{v_{\mathrm{upperarm}}\cdot v_{\mathrm{forearm}}}{\left|\right|v_{\mathrm{upperarm}}\left||\;|\right|v_{\mathrm{forearm}}\left|\right|}\right),$$

and $v_{\mathrm{upperarm}}=\left(e_{x}-s_{x},\;e_{y}-s_{y}\right)$ with $\left(s_{x},s_{y}\right)$ as the shoulder coordinates.

Finally, the medial/lateral rotation angle is estimated as

(11) $$\theta_{\mathrm{rotation}}=\sin^{-1}\;\left(\frac{L_{\mathrm{corrected}}}{L_{\max}}\right),$$

where $L_{\max}$ is the maximum calibrated forearm length during full extension.

### 3.3. System Workflow

**Figure 3:**
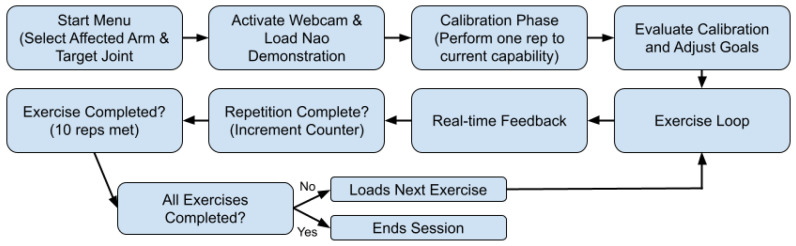
Systemworkflow mapping out the thought process of the system.

Upon initiation, the system presents an intuitive start menu, designed with Tkinter, that enables the patient to select their target limb (left or right arm) and the specific joint (elbow or shoulder) intended for rehabilitation. Following the selection, the system activates either an integrated or external webcam to track the patient’s movements. Concurrently, an instructional video demonstrating the exercise, performed by the NAO robot, is displayed alongside a real-time webcam feed. Clear and informative overlays—including exercise instructions, joint angle measurements, and helpful visual diagrams rendered via OpenCV—provide immediate, actionable feedback, see Figure 3.

The rehabilitation session begins with a calibration phase, wherein the patient is instructed to perform one repetition of the demonstrated exercise at their maximum achievable range. Using the MediaPipe BlazePose pose estimation model, the system records and evaluates joint angles during this initial repetition. Suppose the performed repetition does not reach the full ideal range of motion criteria, defined using clinical sources [41,42]: the system detects the discrepancy and dynamically adjusts the subsequent exercise parameters, ensuring personalized and realistic therapeutic goals that align with the patient’s current capabilities.

Throughout the session, the system continuously captures video frames from the webcam and processes them using the BlazePose model to identify anatomical landmarks accurately. These 2D landmark coordinates enable the precise computation of joint angles through trigonometric and vector calculations. Real-time joint angle measurements are then compared against established ideal ranges, determined during the calibration phase, allowing the system to accurately assess the quality and phase (such as “up” or “down” and “full extension” or “full flexion”) of each executed movement.

Patients perform the exercises guided by the NAO robot demonstration, and clear graphical feedback is displayed on the screen. Instructions and corrective feedback are dynamically overlaid onto the live webcam feed, facilitating immediate corrections and ensuring adherence to the desired movement patterns. Upon successful completion of the required range of motion for each phase, repetitions are automatically counted. Incomplete or incorrect attempts are disregarded, though future developments could record these failed reps to track individual progress throughout multiple sessions. After achieving a set of ten repetitions, the system seamlessly transitions to the subsequent exercise. This calibration and feedback loop is systematically repeated for each prescribed exercise targeting the chosen joint. The rehabilitation session concludes automatically upon the completion of all assigned exercises or manually upon the patient’s request. This adaptive, responsive design allows the system to continuously tailor the therapeutic experience to the patient’s evolving needs, ensuring personalized, effective, and engaging rehabilitation.

## 4. Experimental Setup

### 4.1. Participants and Informed Consent

Thirteen volunteers participated in total, all of whom were healthy adults (A≥B18 years old) with no history of upper limb injuries, neurological disorders, or musculoskeletal impairments, and were gathered through networking within the local community. Inclusion criteria required the full cognitive ability to understand and follow instructions. Individuals with current or past shoulder/elbow pathology, surgical history, or ongoing rehabilitation were excluded. Participant demographics are summarized in Table 2.

Prior to participation, all individuals received a detailed explanation of the study objectives and procedures and any potential risks. Written informed consent was obtained from each participant. This study involved minimal risk, and no identifiable personal data were collected.

### 4.2. Experiment Overview

**Figure 4:**
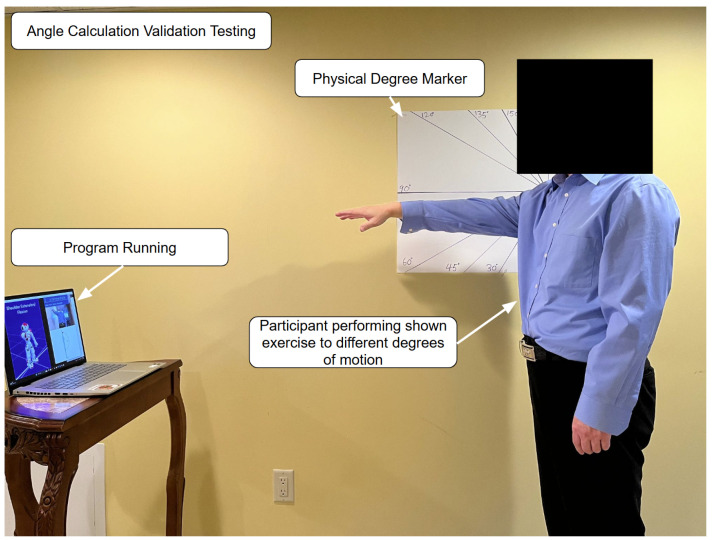
Experimental setup of angle calculation validation testing.

The first experiment, an angle calculation validation testing (shown in Figure 4), was conducted in a quiet, well-lit room with ample space to conduct safe and comfortable upper limb movements. The room contained a single plain-colored flat wall. Clearly visible physical angle markers (30°, 45°, 60°, 90°, 120°, 135°, 150°, and 180°) were drawn on big pieces of paper and pinned onto the wall as visual and measurable guides for correct limb placement.

The rehabilitation setup consisted of a laptop with an ordinary webcam that the rehabilitation program ran on. It was placed onto a stable table in front of the participant at waist height, approximately 1 m from the participant. This configuration ensured the maximum angle coverage and unobstructed visibility of participant movement.

Five volunteer participants aged 18–65, recruited through local networking, all of whom had no history of upper limb injury or mobility impairment, undertook the trials. This sample aims to ensure the required degree of poses are properly executed in line with the physical markers on the wall to eliminate confounding factors.

The participants performed the series of arm exercises, placing their limb precisely in line with each of the prescribed angle markers on the wall. During each trial, the rehab software determined the joint angle in real time using its internal angle calculation functions. Simultaneously, both the visibly confirmed angle from the wall markers and the system-determined angle were recorded for comparison. The recorded system angles for each angle marker were averaged for the participants.

This controlled test aimed to measure the program’s joint angle detection accuracy by pitting its software-calculated angles against conventional physical angle markers. This validation step was undertaken to determine the reliability and accuracy of the program’s real-time feedback mechanisms in clinical and home-based rehabilitation use with the possibility of high occlusion, especially in home-based settings.

**Figure 5:**
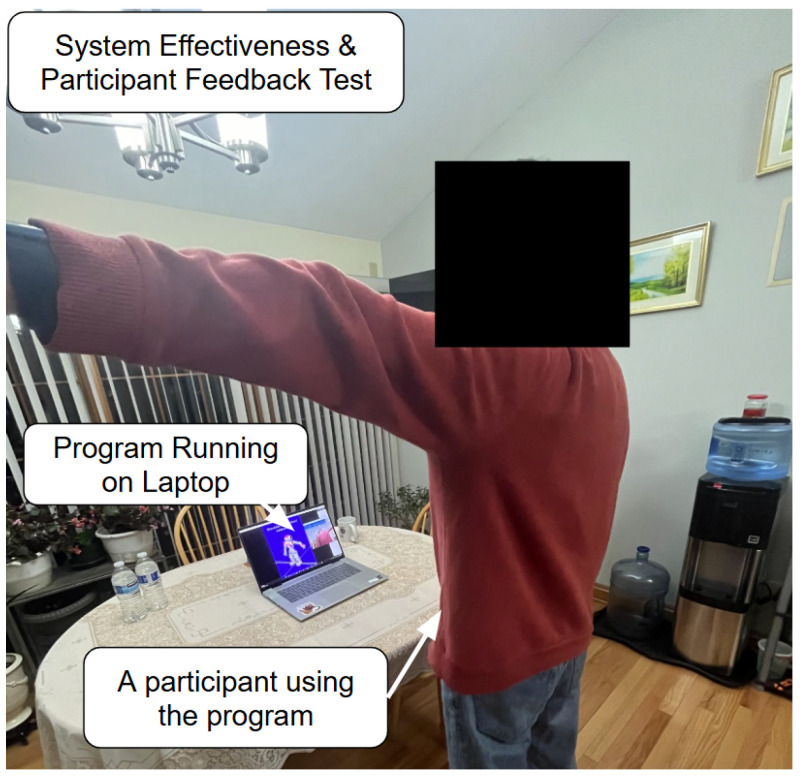
Experimental setup of system effectiveness and participant feedback testing.

For the next experiment (the example shown in Figure 5), the 13 healthy participants, aged 18–65, recruited through networking within the local community, performed all exercises using the system. The trials were conducted inside the participants’ homes in order to effectively emulate the home-based application of the program. The objective was to determine whether the system feedback improved a patient’s exercise accuracy. This is undertaken by splitting the detected cycles into the first half and second half, computing the average range of motion (ROM) for each half, repeating this across all participants and finding the grand average for each half, modeling the average repetitions using sinusoidal functions, calculating their RMSEs (Root Mean Square Errors) against the ideal trajectory, and comparing the calculated error between the first half and second half to determine accuracy improvements. A post-survey was also filled out by all participants which evaluated various aspects of the system based on personal experience.

### 4.3. Data Splitting and Cycle Segmentation

The raw angle data is collected and stored in an array by the program for processing after the session ends.

**Data Acquisition:** The angle data are recorded at uniform time intervals (every 0.5 s).

**Cycle Definition:** Each cycle represents a complete movement from a minimum (baseline) to a maximum (peak) and back to a minimum.

**Trough Detection:** Local minima (troughs) are detected in the data using an algorithm (by applying the find_peaks function on the negative of the data). Each cycle is defined from one trough to the next.

For each cycle,

ROM=Peak−Baseline

where the baseline is the angle at the trough, and the peak is the maximum angle within that cycle.

### 4.4. Splitting into Two Halves and Averaging

The angle data is then split into two halves and each half is averaged.

**Cycle Grouping:** Once cycles are identified using the find_peaks function from SciPy (1.13.0), they are split into two groups: the first half and the second half of the session.

**Averaging:** For each half, compute the average baseline and the average ROM:

$$\bar{\mathrm{Baseline}}=\frac{1}{n}\sum_{i=1}^{n}{\mathrm{Baseline}}_{i},\;\bar{\mathrm{ROM}}=\frac{1}{n}\sum_{i=1}^{n}{\mathrm{ROM}}_{i}$$

This process is repeated for all participants’ data, and the resulting averages are averaged for each half.

### 4.5. Modeling the Average Rep

A normalized time axis is defined from t=0 to t=1 for one complete cycle, where t=0 corresponds to the start of the repetition and t=1 to its completion. Each individual repetition, regardless of its absolute duration, is rescaled to this unit interval so that intermediate points (0<t<1) represent proportional progress through the movement. This normalization allows repetitions of different lengths to be compared on a common temporal basis and enables averaging across participants without being affected by execution speed.

The average movement is modeled using a sinusoidal function:

$$\mathrm{Model}\left(t\right)=\bar{\mathrm{Baseline}}+\bar{\mathrm{ROM}}\cdot \sin\left(\pi t\right)$$

This function assumes that

- At t=0 and t=1, the angle is at the baseline (neutral);
- At t=0.5, the movement reaches the peak.

### 4.6. Ideal Trajectory and RMSE Calculation

The ideal movement is defined as

Ideal(t)=IdealRange·sin(πt)

For example, if the ideal ROM is 180°, then

Ideal(t)=180·sin(πt)

The RMSE between the modeled average rep and the ideal function is calculated as

$$\mathrm{RMSE}=\sqrt{\frac{1}{N}\sum_{i=1}^{N}{\left(\mathrm{Model}\left(t_{i}\right)-\mathrm{Ideal}\left(t_{i}\right)\right)}^{2}}$$

where $t_{i}$ are sample points on the normalized time axis.

This process is repeated across both halves of each exercise.

## 5. Results and Discussion

The results of this study demonstrate that the proposed computer-vision-based rehabilitation system effectively computes the patients’ joint angles and enhances their movement accuracy across a variety of upper limb exercises. The quantitative assessment using the Root Mean Square Error (RMSE) serves as an objective measure of improvement, and the observed trends align with the system’s intended function of guiding and refining patients’ exercise performance. The demographic data of the participants are shown in Table 2.

### 5.1. Angle Calculation Validation

The following tables display the trial results of the first experiment: the angle calculation validation test. Each trial consists of the angle value detected by the system for the corresponding physical angle value averaged across the five participants, which include participants with the IDs HP1–HP5.

Validation results indicate that joint angle detection using the system varied in terms of accuracy levels depending on which movement was being tested. Among the four movements that were tested, the smallest error in terms of the RMSE was for shoulder abduction/adduction (Table 3) at 1.04°, and the largest RMSE was for shoulder flexion/extension (Table 4) at 8.13°. These results suggest that the system performs best by tracking joint movements mostly in the coronal plane, in which joint characteristics are less occluded and angle changes are more apparent for a front-facing camera.

Elbow flexion/extension (Table 5) produced an RMSE of 8.05°, which, although less than that for shoulder flexion/extension, still indicates the presence of detectable deviation from the actual to the detected angles. This may be due to the reduced joint articulation and greater dynamic changes in angles characteristic of elbow motion and challenging for accuracy in 2D detection in some frames.

Shoulder medial/lateral rotation validation (Table 6) yielded an RMSE of 6.44°, indicating moderate accuracy. Inasmuch as the movement often involves minor internal rotations of the upper arm and potential occlusion of elbow or wrist landmarks, the ability of the system to maintain a sub-5° error in this exercise is evidence of error-free rotational movement measurements.

On every task, the estimated errors were well within acceptable limits for non-clinical rehabilitation environments, particularly when the system is used to track trends or identify large changes over time rather than for diagnostics. Overall, employing decimal-level precision in the RMSE measures maintains the evaluation as designed to reflect true detection fidelity, even when rounding to the nearest integer on the system interface for user simplicity.

Overall, quantitative verification demonstrates that the system offers clinically valid accuracy for a variety of upper limb movements, with a particularly strong performance for diverse motions with minimal occlusion and high-contrast vision.

### 5.2. Movement Accuracy Improvements

The following graphs show the modeled function of the detected angle value averages between the 13 participants during the second test compared with the ideal graph, separated into the first half and the second half in order to show the improvement.

**Figure 6:**
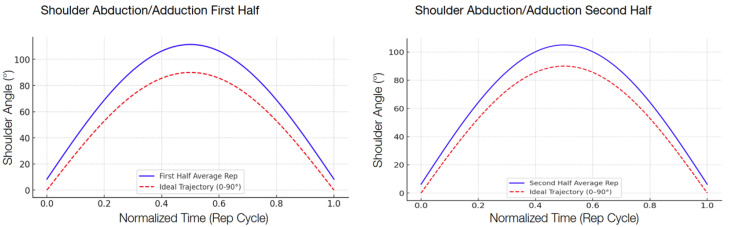
Comparison of shoulder abduction/adduction trajectories. The graphs model the average shoulder range of motion during the first and second halves of the exercise repetitions, averaged across the 13 participants. The solid blue line represents the average participant trajectory, while the dashed red line shows the ideal trajectory from 0° to 90°; comparing the two graphs gives a visual representation of participant accuracy improvements throughout the exercise.

**Figure 7:**
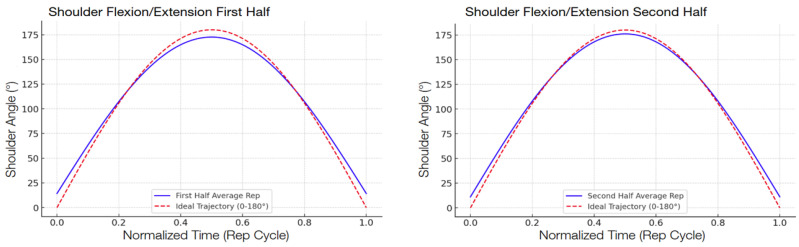
Comparison of shoulder flexion/extension trajectories. The graphs model the average shoulder range of motion during the first and second halves of the exercise repetitions, averaged across the 13 participants. The solid blue line represents the average participant trajectory, while the dashed red line shows the ideal trajectory from 0° to 180°; comparing the two graphs gives a visual representation of participant accuracy improvements throughout the exercise.

**Figure 8:**
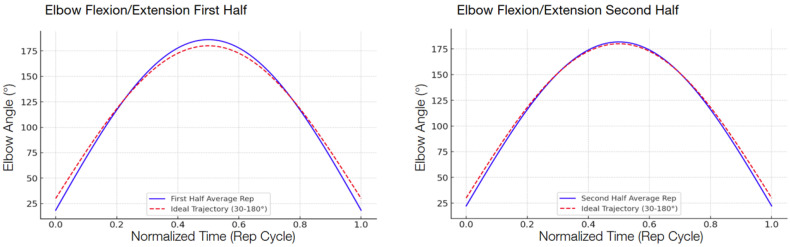
Comparison of elbow flexion/extension trajectories. The graphs model the average elbow range of motion during the first and second halves of the exercise repetitions, averaged across the 13 participants. The solid blue line represents the average participant trajectory, while the dashed red line shows the ideal trajectory from 30° to 150°; comparing the two graphs gives a visual representation of participant accuracy improvements throughout the exercise.

**Figure 9:**
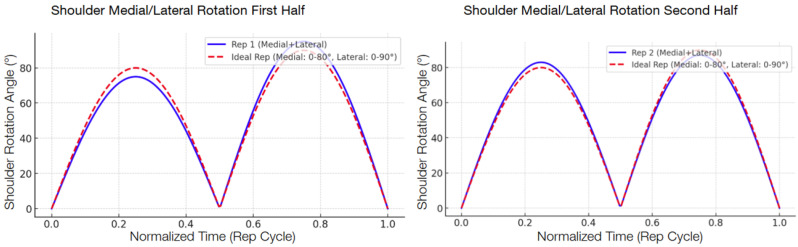
Comparison of shoulder medial/lateral rotation trajectories. The graphs model the average shoulder range of motion during the first and second halves of the exercise repetitions, averaged across the 13 participants. The solid blue line represents the average participant trajectory, while the dashed red line shows the ideal trajectory from 0° to 80° (medial rotation) and 0° to 90° (lateral rotation); comparing the two graphs gives a visual representation of participant accuracy improvements throughout the exercise.

There was a consistent reduction in the RMSE across all four targeted exercises when comparing the first half and the second half of the repetitions, shown in Table 7. The reduction indicates that the participants, by interacting with the system, became increasingly accurate in their movement patterns within a single session. Shoulder abduction/adduction (Figure 6), specifically, indicated a 29.3 percent decrease in the RMSE, from 17.08° to 12.08°. Shoulder flexion/extension (Figure 7) revealed a slightly higher decrease of 32.5 percent, with the RMSE decreasing from 6.80° to 4.59°. Elbow flexion/extension (Figure 8) showed the largest improvement, with a 42.3 percent decrease (from 5.98° to 3.45°), reflecting that the system could handle exercises well for less complex hinge-like joints. Shoulder medial/lateral rotation (Figure 9) also saw a notable RMSE decrease of 39.9 percent, from 3.53° to 2.12°. The different levels of improvement between exercises could correspond to differences in joint complexity and movement visibility in the field of view of the camera.

### 5.3. Interpretation and Clinical Relevance

These results demonstrate that real-time feedback supplied by the computer vision AI system can significantly improve the movement accuracy, achieving the primary research objective. Notably, even more complicated movements, such as shoulder rotation, showed noteworthy error reductions.

This result has significant clinical and home-based rehabilitation implications. Reduction in the RMSE reflects more accurate control of joint movements, which are essential to effective motor recovery. Through encouraging patients toward the proper performance, the system can facilitate compensation maneuver mitigation, injury risk minimization, and regular compliance to rehabilitation programs.

The participants’ response, shown in Table 8, also supports the system’s effectiveness and acceptability. The overall satisfaction was high, as expressed through the survey responses, with most areas of the system receiving high Likert-scale scores. The NAO robot video presentation had slightly lower (though still moderate) scores compared to those for other areas. This suggests that while the system’s functional guidance is good, its use of the NAO robot as a visual exemplar is less effective than originally intended and may potentially be improved by either making the animations more effective or by attempting alternative forms of demonstration.

Notably, the absence of low ratings (scores of 1 or 2) suggests that the system operated at or above user expectations for most participants. The occasional lower ratings likely reflect personal preference rather than systemic problems and could be addressed by adding more customization features such as color schemes, audio instructions, or font adjustments.

## 6. Conclusions

The ability of the system to provide measurable improvements in movement accuracy without requiring expensive equipment, dedicated sensors, or constant professional oversight highlights its potential to expand access to effective rehabilitation. Its affordability, accessibility, and efficacy position it as a suitable option for supplementing home-based rehabilitation, particularly for patients facing economic barriers to traditional therapy.

Nevertheless, important limitations remain. Webcam-based systems are inherently susceptible to occlusions, especially in uncontrolled home environments, which may reduce tracking accuracy. Future work should address this challenge by exploring hybrid solutions that combine vision-based tracking with wearable or depth-sensing technologies. In particular, inertial measurement units (IMUs) have been shown to enhance motion tracking accuracy in rehabilitation contexts [43]. Kinect-based methods for investigating joint movement connections have also demonstrated promising results [44,45,46] and could be adapted to improve accuracy and robustness in our framework. However, these will be optional, as the additional expensive hardware can be unaffordable to some.

Further developments will also include expanding the system to support more complex compound movements, implementing bilateral execution of motor tasks (e.g., shoulder flexion) to better reflect real-life functionality, and integrating robotic assistance when required for impaired users. Moreover, while incomplete or incorrect repetitions are excluded from sessions currently, future versions of the system could log such attempts as valuable indicators of user progress, enabling longitudinal tracking and adaptive feedback during rehabilitation. Additionally, features such as personalized exercise plan generation and visual customization can further improve flexibility and adaptability across diverse patient populations.

## Figures and Tables

**Table 1:** Comparison of representative telerehabilitation systems based on sensing modality, guidance, feedback, and key outcomes.

Study (Year)	Modality/Sensing	Guidance Modality	Automated Feedback	Setting and Population (N)	Key Outcome(s)
Adolf et al. [25] (2022)	Single RGB webcam; OpenPose 2D HPE	N/A (tracking subcomponent)	Keypoint confidence; posture effects	Home-exercise video dataset (>2000 unique exercises)	Demonstrated robustness of webcam HPE for home exercises; side and floor positions reduced accuracy; occlusion and lower-body joints most affected.
Cóias et al. [28] (2022)	Laptop webcam; markerless HPE	Virtual coach (on-screen)	RT audiovisual cues; compensation (trunk) detection	Stroke rehab; system evaluation on videos (15 post-stroke) and usability (7 volunteers)	Corrects excessive trunk compensation during reaching; low-cost, home-oriented workflow reported as usable.
Nakano et al. [20] (2020)	Multi-camera RGB (five cams) and OpenPose 2D → 3D DLT	N/A (measurement subcomponent)	3D joint positions; accuracy vs. optical mocap	Lab; two healthy adults; walking/jump/throw tasks	∼80% of joint-position MAEs <30 mm; errors driven by 2D tracking failures; shows path to accurate markerless 3D with consumer cameras.
Ota et al. [26] (2020)	Single camera and OpenPose vs. Vicon	N/A (validation subcomponent)	Joint angles (trunk/hip/knee/ankle) during squats	Lab; 20 healthy adults	Reported reliability of OpenPose angles vs. Vicon for bilateral squat; supports feasibility of low-cost video for clinic assessments.
Osawa et al. [32] (2023)	Monocular RGB; OpenPose and 3D reconstruction	On-screen guidance	DTW-based motion similarity scoring; RT feedback	Home-oriented design (whole-body exercises)	Low-cost, single-camera telerehab with 3D pose and similarity evaluation; designed for intuitive remote coaching without wearables.
Clemente et al. [33] (2024)	MediaPipe Pose (3D from monocular 2D)	N/A (measurement subcomponent)	ROM estimation across eight physio exercises; accuracy vs. ground truth	Lab; musculoskeletal physio tasks	ROM MAPE ∼15–25% with high correlations; highlights strengths (e.g., shoulder abduction) and limits (occlusion/depth).
Assad-Uz-Zaman et al. [29] (2021)	Kinect V2 depth and NAO	NAO humanoid	NAO mirrors therapist’s joint angles (IK)	Lab; feasibility (demo with adult operator)	Demonstrated therapist-driven, remote NAO instruction for upper limb rehab movements; shows social-robot guidance feasibility.
Raso et al. [30] (2024)	Vision-guided NAO	NAO humanoid (scripted demos)	Temporal pacing; ROM and smoothness indices	Pilot: 10 healthy and 2 with shoulder pathology	NAO guidance improved temporal control and smoothness during shoulder tasks; supports robot-coach engagement benefits.
Komaris et al. [34] (2022)	Single IMU (wearable)	Video demo; no robot	Unsupervised at-home performance metrics (smoothness, intensity, regularity)	30 healthy adults; lab and at-home week	IMU features sensitive to at-home execution quality; showed need for feedback pacing; objective adherence tracking without video.

**Table 2:** Demographic data of the participants.

Participant ID	Occupation	Age Range	Gender	Dominant Hand	Race
HP1	E	64+	M	R	AS
HP2	U	50–64	M	R	AS
HP3	E	46–50	F	R	AS
HP4	R	50–64	F	L	AS
HP5	E	50–64	M	R	AS
HP6	E	50–64	M	R	AS
HP7	U	18–24	M	L	HI
HP8	U	18–24	F	R	AS
HP9	E	46–50	F	R	AS
HP10	U	18–24	M	R	NHW
HP11	U	18–24	M	R	AS
HP12	E	25–34	M	R	AS
HP13	R	35–40	M	R	AS

Abbreviations: Participant ID: HP = Healthy Participant; Occupation: E = Employed, U = Unemployed, R = Retired; Gender: M = Male, F = Female; Dominant Hand: R = Right Arm, L = Left Arm; Race: AS = Asian, AA = African American, HI = Hispanic, NHW = Non-Hispanic White.

**Table 3:** Shoulder abduction/adduction validation testing results.

Trial	Known (°)	Detected (°)	Error (°)	Squared Error
1	30	30.3	+0.3	0.09
2	45	43.5	−1.5	2.25
3	60	59.8	−0.2	0.04
4	90	88.6	−1.4	1.96
**RMSE across all trials:**				**1.04°**

**Table 4:** Shoulder flexion/extension validation testing results.

Trial	Known (°)	Detected (°)	Error (°)	Squared Error
1	30	27.0	−3.0	9.00
2	45	43.1	−1.9	3.61
3	60	57.6	−2.4	5.76
4	90	90.8	+0.8	0.64
5	120	132.8	+12.8	163.84
6	135	151.3	+16.3	265.69
7	150	158.5	+8.5	72.25
8	180	177.1	−2.9	8.41
**RMSE across all trials:**				**8.13°**

**Table 5:** Elbow flexion/extension validation testing results.

Trial	Known (°)	Detected (°)	Error (°)	Squared Error
1	30	19.3	−10.7	114.49
2	45	33.1	−11.9	141.61
3	60	49.5	−10.5	110.25
4	90	92.6	+2.6	6.76
5	120	123.9	+3.9	15.21
6	135	143.0	+8.0	64.00
7	150	148.8	−1.2	1.44
**RMSE across all trials:**				**8.05°**

**Table 6:** Shoulder medial/lateral rotation validation testing results.

Trial	Known (°)	Detected (°)	Error (°)	Squared Error
1	30	38.1	+8.1	65.61
2	45	53.5	+8.5	72.25
3	60	65.3	+5.3	28.09
4	90	89.8	−0.2	0.04
**RMSE across all trials:**				**6.44°**

**Table 7:** RMSE comparisons between the first and second half.

Exercise	RMSE First Half	RMSE Second Half	RMSE % Decrease
Shoulder Abduction/Adduction	17.08°	12.08°	29.3%
Shoulder Flexion/Extension	6.80°	4.59°	32.5%
Elbow Flexion/Extension	5.98°	3.45°	42.3%
Shoulder Medial/Lateral Rotation	3.53°	2.12°	39.9%

**Table 8:** Post-experiment participant survey ratings for system attributes (Likert scale: 1 = lowest, 5 = highest). Values represent the number of participant responses for each rating.

Attribute	Rating					Mean Score
	5	4	3	2	1	
Effectiveness	9	2	1	1	0	4.46
Functionality	9	2	0	1	1	4.38
Flexibility	9	3	1	0	0	4.62
Safety	10	1	2	0	0	4.62
Comfortability	10	0	2	1	0	4.54
Clarity	9	4	0	0	0	4.69
Exercise Selection	9	3	1	0	0	4.62
Video Demonstration	9	3	0	1	0	4.54
Ease of Use	10	2	0	0	1	4.54

## Data Availability

The data that support the findings of this study are available from the corresponding author [Kevin Hou] upon reasonable request.
